# Population history, phylogeography, and conservation genetics of the last Neotropical mega-herbivore, the lowland tapir (*Tapirus terrestris*)

**DOI:** 10.1186/1471-2148-10-278

**Published:** 2010-09-14

**Authors:** Benoit de Thoisy, Anders Gonçalves da Silva, Manuel Ruiz-García, Andrés Tapia, Oswaldo Ramirez, Margarita Arana, Viviana Quse, César Paz-y-Miño, Mathias Tobler, Carlos Pedraza, Anne Lavergne

**Affiliations:** 1 Association Kwata, BP 672, 97335 Cayenne cedex, French Guiana; 2 Laboratoire des Interactions Virus-Hôtes, Institut Pasteur de la Guyane, Cayenne, French Guiana; 3 CSIRO Marine and Atmospheric Research, GPO Box 1538, Hobart, TAS 7001, Australia; 4 Laboratorio de Genética de Poblaciones Molecular-Biología Evolutiva, Facultad de Ciencias, Pontificia Universidad Javeriana, Bogotá DC., Colombia; 5 Centro Tecnológico de Recursos Amazónicos, Centro Fátima, Casilla 16-01-800 Puyo-Pastaza, Ecuador; 6 Programa de Maestría en Biodiversidad de Áreas Tropicales y su Conservación MBATC, CSIC/UCE/UIMP, Quito, Ecuador; 7 Unidad de Biología Integrativa, Laboratorios de Investigación y Desarrollo, Facultad de Ciencias y Filosofía, Universidad Peruana Cayetano Heredia, Lima, Perú; 8 Facultad de Ciencias Veterinarias de Esperanza, Universidad Nacional del Litoral, Buenos Aires, Argentina; 9 Instituto de Investigaciones Biomédicas, Universidad de las Américas, Avenida Granados y Colimes, Quito, Ecuador; 10 Andes to Amazon Biodiversity Program, Botanical Research Institute of Texas, Fort Worth, TX 76102, USA; 11 Laboratorio de Biología Evolutiva de Vertebrados, Departamento de Ciencias Biológicas, Universidad de Los Andes, Bogota, Colombia

## Abstract

**Background:**

Understanding the forces that shaped Neotropical diversity is central issue to explain tropical biodiversity and inform conservation action; yet few studies have examined large, widespread species. Lowland tapir (*Tapirus terrrestris*, Perissodactyla, Tapiridae) is the largest Neotropical herbivore whose ancestors arrived in South America during the Great American Biotic Interchange. A Pleistocene diversification is inferred for the genus *Tapirus *from the fossil record, but only two species survived the Pleistocene megafauna extinction. Here, we investigate the history of lowland tapir as revealed by variation at the mitochondrial gene Cytochrome *b*, compare it to the fossil data, and explore mechanisms that could have shaped the observed structure of current populations.

**Results:**

Separate methodological approaches found mutually exclusive divergence times for lowland tapir, either in the late or in the early Pleistocene, although a late Pleistocene divergence is more in tune with the fossil record. Bayesian analysis favored mountain tapir (*T. pinchaque*) paraphyly in relation to lowland tapir over reciprocal monophyly, corroborating the inferences from the fossil data these species are sister taxa. A coalescent-based analysis rejected a null hypothesis of allopatric divergence, suggesting a complex history. Based on the geographic distribution of haplotypes we propose (*i*) a central role for western Amazonia in tapir diversification, with a key role of the ecological gradient along the transition between Andean subcloud forests and Amazon lowland forest, and (*ii*) that the Amazon river acted as an barrier to gene flow. Finally, the branching patterns and estimates based on nucleotide diversity indicate a population expansion after the Last Glacial Maximum.

**Conclusions:**

This study is the first examining lowland tapir phylogeography. Climatic events at the end of the Pleistocene, parapatric speciation, divergence along the Andean foothill, and role of the Amazon river, have similarly shaped the history of other taxa. Nevertheless further work with additional samples and loci is needed to improve our initial assessment. From a conservation perspective, we did not find a correspondence between genetic structure in lowland tapir and ecogeographic regions proposed to define conservation priorities in the Neotropics. This discrepancy sheds doubt into this scheme's ability to generate effective conservation planning for vagile species.

## Background

A central debate in Neotropical biogeography concerns the importance of Pleistocene climatic and geological events in generating current species richness [1-4]. Molecular evidence across several taxa supports a scenario where most of the observed divergence and speciation events occurred prior to the Pleistocene [5], and are associated with paleogeographic events of the late Miocene [6,7]. More recent studies, however, suggest a more significant role to the climatic cycles of the Pleistocene than what was previously hypothesized [4]. In this paper we contribute to this debate by exploring the phylogeography and population history of the largest terrestrial Amazonian mammal, the lowland tapir (*Tapirus terrestris*) whose genus has a history in South America confined to the Pleistocene.

Tapirs (Perissodactyla, Tapiridae, *Tapirus*) were part of a community of large Neotropical browsers that largely disappeared at the end of the Pleistocene [8-10]. As the last representative of this community, tapirs are key actors in forest dynamics as functionally important seed dispersers and predators [11,12], often being called forest "gardeners" or "architects" [13]. The genus *Tapirus *has four extant species, the Malay tapir (*T. indicus*) in South Asia, and three Neotropical species: the mountain tapir (*T. pinchaque*) in the Andes' central mountains, the Baird's tapir (*T. bairdii*) in Central America; and the lowland tapir (*T. terrestris*) occupying the widest distribution, from Venezuela to northern Argentina, and from the Brazilian Atlantic forest to the Ecuadorian sub-Andean foothills, and variety of habitats (e.g. moist and swamp forests, dry and moist woodlands, savannas, and a wide range of wetlands) [14].

Current knowledge on lowland tapir evolutionary history is based on a well-studied fossil record for the southern part of its range [15-17] but a more sparsely explored fossil record in the Amazon region. Moreover, only a single study focused on estimating dates of *Tapirus *divergence using sequence variation at the mitochondrial cytochrome oxidase II gene [18]. Inferences from the fossil record indicate that the genus became widespread in the Nearctic by the early Pleistocene [8], and migrated into South America between 3.1 to 2.7 million years before present (My BP) during the Great American Biotic Interchange [19-21]. The earliest South American species, *T. merriami*, is recorded in the Marplatan South American Land Mammal Age (SALMA) in the Pliocene [22]. Then during the Ensenadan SALMA, a number of other *Tapirus *species co-occured in the fossil record, suggesting a wave of diversification within South American [15,17,23-26]. This pattern follows into the Lujanian SALMA, when the genus likely experienced a second period of diversification, with several species occurring sympatrically: *T. olivaresi*, *T cristatellus*, *T. mesopotamicus*, *T. rioplatensis*, *T. tarijensis *[27]. The earliest fossil records of *T. terrestris *are reported at this period (80-130 Ky BP), in the Mesopotamian region of northern Argentina [16,24,28,29]. Other records are more recent, in southwestern Brazilian Amazonia (30-45 Ky BP [15]) and in northern Uruguay (6-15 Ky BP [17]).

Morphological data suggest that *T. terrestris*, *T. pinchaque*, and *T. mesopotamicus *(extinct) form a monophyletic group, and *T. bairdii *forms a monophyletic group with North American *Tapirus *extinct species [24]. Thus, the fossil record in combination with morphological analyses indicate that *Tapirus *diversified within South America during the past 2.5 My BP, similarly to other recently arrived North American taxa [21], with *T*. *terrestris *likely emerging in the late Pleistocene. Two other inferences can be made from the fossil record. First, as in other lowland Amazonia species [e.g., [30]], the lowland tapir's origin of diversification and spread is likely western Amazonia, where fossils of several distinct *Tapirus *species (of which only *T. terrestris *remains), are purported to have occurred in sympatry in the past 100 Ky BP [15], indicating this region's important role in Neotropical diversification during the mid to late Pleistocene. Second, the earliest fossil records of lowland tapir are reported for the late Pleistocene in the southern range of the genus' current distribution [19] indicating the range expansion of the lowland tapir was rapid.

Several mechanisms of diversification have been proposed for the Pleistocene [31], leading to either allopatric or parapatric divergence. The Refuge hypothesis proposes that a series of alternating climatic changes caused by Croll-Milankovitch cycles combined with the Andes and other higher elevation terrains to allow for the formation of humid forest refugia interspersed by areas of dry forest and open grasslands. Within the refuges, lowland forest dwelling species remained isolated, creating an opportunity for allopatric speciation, evidenced in current centers of species endemism [32-34]. Such refuges during times of climate cooling have been shown to account for divergence in other parts of the world [e.g., [35]], and the hypothesis is one of the most commonly tested in biogeographic studies in Amazonia [e.g., in monkeys: [36,37]]. However, the existence of rainforest refugia in Amazonia is controversial [38,39], and centers of endemism, do not match well across taxa [40]. In addition, allopatric divergence in rainforest refugia does not seem a likely mechanism to explain divergence of the lowland tapir as the species' distribution ranges across South America [13] including large areas of savanna [31]. Allopatric divergence within Amazonia has also been hypothesized to occur as a result of restricted gene flow across large rivers within the Amazon basin. This "river-barrier" model predicts independent lineages occurring on opposite banks as a result of restricted gene flow [41,42]. Geological data suggest that the Amazon River attained its current flow during the late Pleistocene [43], and molecular evidence proposes that the Amazon River has been a significant barrier for several taxa, including monkeys [37] and carnivores [44,45]. Under this scenario the river would be expected to separate independent lowland tapir lineages on either side of its banks.

Finally, it is possible that divergence occurred in parapatry rather than allopatry [5]. The gradient hypothesis proposes that divergence can occur across steep environmental gradients without physical barriers to gene flow and predicts that sister lineages occur in adjacent habitats [46]. In Amazonia, specific tests have rejected the gradient hypothesis in rodents [47] and in birds [48]. Nevertheless, the Andean foothills provide the sharp change in ecotone assumed by the gradient hypothesis, a feature that has been used to explain why the region is a hotspot for diversification in the absence of geographic barriers [49]. In the case of the *Tapirus *genus, the occurrence of mountain and lowland tapirs in adjacent habitats along the Andes elevational gradient [50] indicate that a gradient model of diversification is possible [51]. To test this hypothesis, it is necessary to demonstrate that these species are sister taxa [47].

Here, we examined genetic variation at Cytochrome b (*cytb*) to investigate lowland tapir *Tapirus terrestris *demographic and population history. We examine how molecular information regarding the early history of the genus can explain variability in the fossil data and contribute to the understanding of diversification processes in the Neotropics. The lowland tapir is also an increasingly threatened species [52], with a distribution that includes several ecogeographic units commonly used to identify priority areas for biodiversity conservation [53]. To date, it is unclear how well this conservation scheme reflects tapir history and the likely success of conservation efforts for different tapir lineages. In this regard, we apply coalescent approaches to: (*i*) determine the most likely timing of divergence among lowland tapir *cytb *lineages; (*ii*) determine population structure of lowland tapir *cytb *and explore the paleogeographic events that may have contributed to this structure; (*iii*) investigate the species' demographic history; and (*iv*) examine how the inferred evolutionary history is currently reflected in areas of conservation priority.

## Results

### Sequence variation, phylogenic relationships and dating divergence

**Figure 1:**
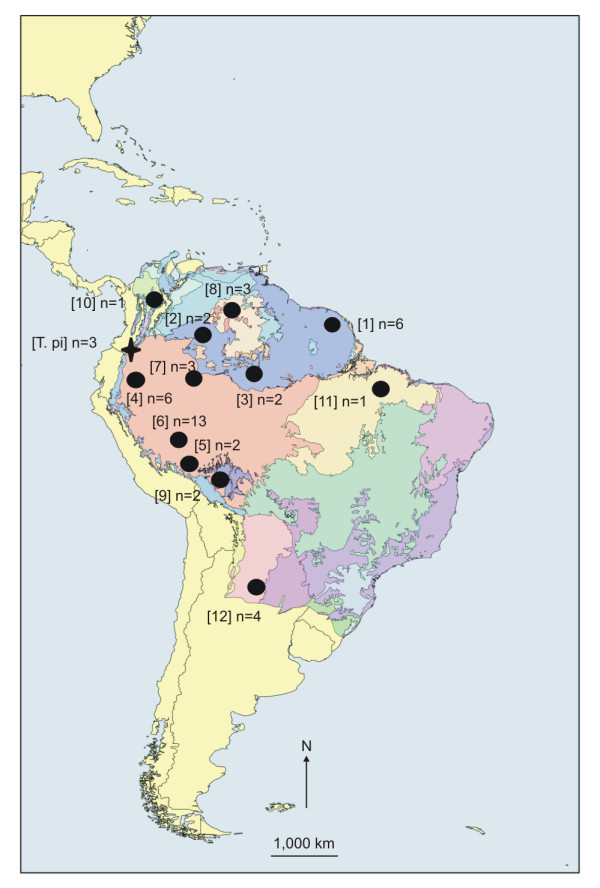
**Lowland tapir (*Tapirus terrestris*) and mountain tapir (*T. pinchaque*) sampling sites**. Indicated are sample size per site, and ecogeographic regions for each sampling site [13]. Numbers are referenced in Table 1.

A total of 1,068 unambiguous bases of the *cytb *gene were sequenced from three mountain tapirs from the Central Andes, Colombia, and 45 lowland tapirs widely sampled across the species distribution range (Figure 1). In lowland tapir sequences, we observed 64 polymorphic sites (61 transitions and 3 transversions), and a bias against guanidine (C: 28.59%, T: 28.66%, A: 29.79%, G: 12.97%). Thirty-five haplotypes were identified with a mean pairwise difference of 0.995%, *H_d_*of 0.988 ± 0.007, and *π *of 0.009325 ± 0.004825. Haplotypes Hte 2 and Hte 13 were found at more than one sampling site: French Guiana and Bolivia, and Peru and Colombia/Brazil border, respectively (Table 1). Finally, two polymorphic sites (defining two haplotypes) were observed among the three mountain tapir sequences.

**Table 1:** Sample identification, geographic origin and ecogeographic unit [13], and associated haplotypes with their respective frequencies

Sample	Origin*	Geographic group	Ecogeographic unit	Haplotype	Frequency
*Tapirus terrestris*					
TG01, TG06, TG29	French Guiana [1]	North Amazon	North-East Amazon rainforest	Hte 1	3/45
TG28	French Guiana [1]	North Amazon	North-East Amazon rainforest	Hte 2	2/45
TG5	French Guiana [1]	North Amazon	North-East Amazon rainforest	Hte 3	1/45
TG24	French Guiana [1]	North Amazon	North-East Amazon rainforest	Hte 7	1/45
TC54	East Colombia [2]	North Amazon	North-East Amazon rainforest	Hte 11	1/45
TC186	East Colombia [2]	North Amazon	North-East Amazon rainforest	Hte 19	1/45
TB01	Brazil, north [3]	North Amazon	North-East Amazon rainforest	Hte 14	1/45
TB02	Brazil, north [3]	North Amazon	North-East Amazon rainforest	Hte 15	1/45
TE17, TE22	Ecuador [4]	Andean Foothill	Upper Amazon rainforest	Hte 4	2/45
TE14	Ecuador [4]	Andean Foothill	Upper Amazon rainforest	Hte 5	1/45
TE19	Ecuador [4]	Andean Foothill	Upper Amazon rainforest	Hte 6	1/45
TE20	Ecuador [4]	Andean Foothill	Upper Amazon rainforest	Hte 8	1/45
TE16	Ecuador [4]	Andean Foothill	Upper Amazon rainforest	Hte 9	1/45
TP104	Peru, south-east [5]	West Amazon	Upper Amazon rainforest	Hte 12	1/45
TP94	Peru, south-east [5]	West Amazon	Upper Amazon rainforest	Hte 16	1/45
TP14	Peru, east [6]	Andean Foothill	Upper Amazon rainforest	Hte 13	2/45
TP4	Peru, east [6]	Andean Foothill	Upper Amazon rainforest	Hte 23	1/45
TP12, TP13	Peru, east [6]	Andean Foothill	Upper Amazon rainforest	Hte 24	2/45
TP11	Peru, east [6]	Andean Foothill	Upper Amazon rainforest	Hte 25	1/45
TP9	Peru, east [6]	Andean Foothill	Upper Amazon rainforest	Hte 26	1/45
TP6, TP7	Peru, east [6]	Andean Foothill	Upper Amazon rainforest	Hte 27	2/45
TP5, TP2	Peru, east [6]	Andean Foothill	Upper Amazon rainforest	Hte 28	2/45
TP3	Peru, east [6]	Andean Foothill	Upper Amazon rainforest	Hte 29	1/45
TP1	Peru, east [6]	Andean Foothill	Upper Amazon rainforest	Hte 30	1/45
TP10	Peru, east [6]	Andean Foothill	Upper Amazon rainforest	Hte 31	1/45
TC88	Colombia/Brazil frontier [7]	West Amazon	Upper Amazon rainforest	Hte 18	1/45
TC68	Colombia/Brazil frontier [7]	West Amazon	Upper Amazon rainforest	Hte 20	1/45
TB69	Colombia/Brazil frontier [7]	West Amazon	Upper Amazon rainforest	Hte 13	2/45
TV95, TV48, TV46	Venezuela [8]	North Amazon	Llanos	Hte 10	3/45
TBo183	Bolivia [9]	South Amazon	"Pastizal" herbaceous habitat	Hte 2	2/45
TBo85	Bolivia [9]	South Amazon	"Pastizal" herbaceous habitat	Hte 22	1/45
TC100	Colombia, west [10]	West Amazon	Choco Darien rainforest	Hte 17	1/45
TB29	Brazil, south of the Amazon mouth [11]	South Amazon	South East Amazon rainforest	Hte 21	1/45
TA10	Argentina [12]	South Amazon	Dry tropical forest	Hte 32	1/45
TA11	Argentina [12]	South Amazon	Dry tropical forest	Hte 33	1/45
TA12	Argentina [12]	South Amazon	Dry tropical forest	Hte 34	1/45
TA13	Argentina [12]	South Amazon	Dry tropical forest	Hte 35	1/45
					
*Tapirus pinchaque*					
TPI1, TPI2	Colombia [T. pi]			Hpi 1	2/3
TPI4	Colombia [T. pi]			Hpi 2	1/3

* refers to [site number] on Figure 1.

**Figure 2:**
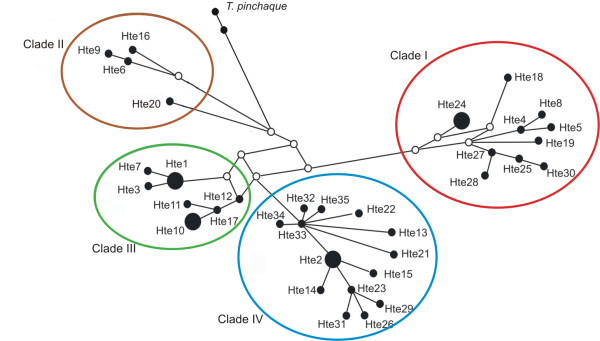
**Minimum spanning network of lowland and mountain tapirs mtDNA Cytochrome b haplotypes**.

**Figure 3:**
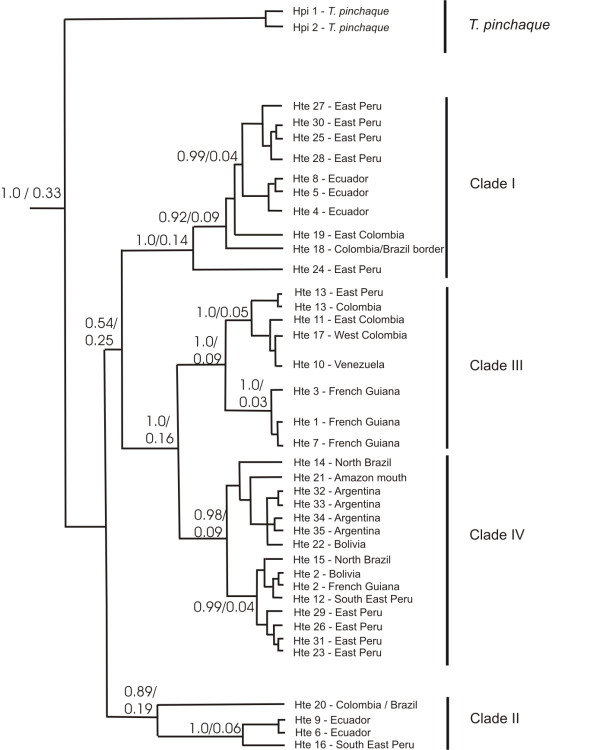
**Bayesian phylogenetic tree and divergence dates, using Ho et al.'s method **[63]. Tip labels refer to haplotype identification number and origin (Table 1). Values above branch nodes refer to posterior probability/time of divergence in My BP.

The Median Joining haplotype network (Figure 2), and the gene genealogies inferred by maximum parsimony, maximum likelihood, and Bayesian coalescent approach (Figure 3) consistently inferred a four-clade structure. In the Bayesian approach, all four clades had posterior probability values > 0.9, and all clades are represented in the strict consensus trees derived from maximum parsimony and maximum likelihood inferences. Clade I groups haplotypes found in western Amazonia: southeast Peruvian Amazon, Ecuadorian Amazon, Colombian Amazon and western Brazilian Amazon. Clade II, similar to clade I, includes haplotypes sampled in the Ecuadorian and Colombian Amazon, eastern Peru, and eastern Colombia. Clade III clusters haplotypes from north Amazonia: northern Atlantic Colombia, Venezuelan and Colombian llanos and French Guiana. Clade IV includes all haplotypes found from south Amazonia including Brazil (except western Amazonia), Argentina, and Bolivia, and some from eastern Peruvian Amazon. Clade IV had the widest geographic distribution, with haplotypes occurring in three of our four defined geographic regions (see Methods). Finally, there was little support for reciprocal monophyly of lowland and mountain tapirs. In the haplotype network, the mountain tapir haplotypes grouped with clade II, and in the Bayesian analyses, the hypothesis that mountain tapirs have a basal position had a posterior probability of 0.26.

Using a Bayesian inference framework, we took two approaches to measure the time of divergence among the four identified clades. In the first approach we used fossil data to calibrate the molecular clock with the timing of the Rhinocerontidae and Tapiridae split, and a well know split within Rhinocerontidae. The inferred mean substitution rate was 5.6 × 10^-3 ^substitutions/site/million years, leading to the inference that divergence between the Asian and the two South American tapir species occurred earlier than the Pliocene (median 19.26 My BP, 95% Highest Posterior Probability: 8.4-35.1 My BP). All clades within lowland tapir are estimated to have diverged some time between the mid-Pliocene and the mid-Pleistocene. In particular, clade I is estimated to have diverged between 0.8 and 4.0 My BP (median = 2.12 My BP, 95%: 0.8-4.0), followed by the split between clade II and clade III (median = 1.5 My BP, 95% HPD: 0.8-4.0), and clade IV between 0.6 and 4.3 My BP (median = 2.0 My BP, 95% HPD: 0.6-4.3). Within the four clades, only a few subclades were strongly supported, with divergence events ranging from mid to late Pleistocene (95% HPD: 0.2-1.4 My BP). In the second analysis, we set a strong prior on the mutation rate based on estimates for Perissodactyl *cytb *mutation rates [54]. This approach inferred a mean substitution rate of 2.5×10^-2 ^substitutions/site/million years (95% HPD: 1.6-3.5 × 10^-2^), which leads to an estimate of *tMRCA *of lowland tapir in the mid to late Pleistocene (median = 0.33 My BP, 95% HPD: 0.19-0.57 My BP). Under this scenario, the split between clades I and II occurred before the start of the last Glacial Maximum (LGM) (medium = 0.25 My BP, 95% HPD: 0.1-0.4); while clades III and IV are estimated to have emerged more recently, possibly during the LGM (median = 0.16 My BP, 95% HPD: 0.08-0.14 My BP).

### Population structure and diversity

We tested the partitioning of genetic variation across three different sampling groupings according to: (*1*) the four phylogenetic clades, as outlined above; (*2*) four geographic regions: the Andean foothills, western Amazonia, south Amazonia and north Amazonia, which largely mirror the geographic distribution of haplotypes within clades; and, (*3*) ecoregions (Figure 1). Using AMOVA, we found significant structuring of genetic variation for all three hypotheses of population structuring (Table 2). In the first grouping, 67.9% of the observed variation was found among groups (*Φ_CT_*= 0.68, *p *≤ 0.0001), and resulted in significant (*p *≤ 0.001) population differentiation across all pairwise comparisons with indices ranging from 0.55 (clade III vs. clade IV) to 0.66 (clade I vs. clade IV; and clade I vs. clade II). Gene and nucleotide diversities varied among these four clades, with lower nucleotide diversity values recorded for clades III and IV (Table 3). Grouping samples by the four geographic regions found 25.53% of the observed variation among groups (*Φ_CT_*= 0.255, *p *≤ 0.0001) (Table 2). Indices of nucleotide diversity were greatest in the two groups from the western Amazonia region: Andean foothills and western Amazonia (Table 3). The south Amazonia group had significant pairwise population differentiation indices (*p *< 0.001) to all other groups, and the same was observed between north Amazonia and Andean foothills groups. Finally, similar levels of among-group variation to those recorded for geographic grouping were observed when partitioning by ecoregions (*Φ_CT_*= 0.246, *p *≤0.001) (Table 2). Nucleotide diversity estimates also varied among ecoregion units, with the highest diversity found in the rainforest ecoregion (Table 3). In contrast, although low levels of genetic structure were observed among most of sampling sites, populations from the Argentinean dry tropical forest and from the Venezuelan llanos were significantly isolated from populations in other ecoregions (Table 3).

**Table 2:** Pairwise *Φ*-statistics among samples grouped according to the phylogenetic clades, the geographic regions, and the ecogeographic regions.

*Phylogenetic clades*							
	Clade I	Clade II	Clade III	Clade IV			
Clade I	-	0.68	0.72	0.72			
Clade II	*p *< 0.001	-	0.64	0.69			
Clade III	*p *< 0.001	*p *< 0.001	-	0.55			
Clade IV	*p *< 0.0001	*p *< 0.001	*p *< 0.001	-			
							

							

*Geographic regions*							
	western Amazonia	Andean foothills	north Amazonia	south Amazonia			
western Amazonia	-	0.08	0.05	0.27			
Andean foothills	ns	-	0.27	0.34			
north Amazonia	ns	*p *< 0.001	-	0.25			
south Amazonia	*p *< 0.01	*p *< 0.01	*p *< 0.01	-			

							

*Ecogeographic regions*							
	North-East Amazon rainforest	"Pastizal" herbaceous habitat	Llanos	Choco Darien rainforest	South East Amazon rainforest	Upper Amazon rainforest	Dry tropical forest
North-East Amazon rainforest	-	0.09	0.22	-0.36	0.13	0.23	0.19
"Pastizal" herbaceous habitat	ns	-	0.80	0.33	0.2	0.23	0.15
Llanos	*p *= 0.01	ns	-	1.0	1.0	0.31	0.84
Choco Darien rainforest	ns	ns	ns	-	1.0	-0.02	0.68
South East Amazon rainforest	ns	ns	ns	ns	-	0.6	0.60
Upper Amazon rainforest	*p *= 0.001	*p *= 0.05	*p *= 0.002	ns	ns	-	0.29
Dry tropical forest	*p *= 0.05	ns	*p *= 0.02	ns	ns	*p *= 0.003	-

Above diagonal: Fixation index (Kimura 2-parameters). Below diagonal: associated *p*-values.

**Table 3:** Genetic diversity estimates and deviation from equilibrium for each of three samples groupings: phylogenetic clades, geographic regions, and ecogeographic units.

Grouping (sample size)	**Haplotype diversity (*H***_***d***_**)**	Nucleotide diversity (*π*)	Tajima's D and Fu's F, and associated *p *values
Phylogenetic clades*			
Clade I (n = 14)	0.956 +/- 0.038	0.004 +/- 0.002	D = -0.94 (ns)/F = -3.01 (*p *= 0.05)
Clade II (n = 4)	1.000 +/- 0.177	0.006 +/- 0.004	D = -0.37 (ns)/F = -0.12 (ns)
Clade III (n = 12)	0.894 +/- 0.063	0.003 +/- 0.002	D = 0.57 (ns)/F = -1.33 (ns)
Clade IV (n = 15)	0.991 +/- 0.028	0.003 +/- 0.002	D = -1.55 (*p *= 0.05)/F = -11.26 (*p *= 0.0001)
			
*Geographic regions*			
western Amazonia (n = 6)	1.000 +/- 0.096	0.010 +/- 0.002	D = -0.87 (ns)/F = -0.77 (ns)
Andean Foothill (n = 19)	0.976 +/- 0.023	0.009 +/- 0.001	D = -0.05 (ns)/F = -3.32 (ns)
north Amazonia (n = 13)	0.923 +/- 0.057	0.006 +/- 0.0005	D = -1.00 (ns)/F = -0.81 (ns)
south Amazonia (n = 7)	1.000 +/- 0.006	0.002 +/- 0.0005	D = -1.61 (*p *= 0.01)/F = -4.56 (*p *= 0.001)
			
*Eco-geographic regions*			
North-East Amazon rainforest (n = 10)	0.933 +/- 0.077	0.007 +/- 0.004	D = -0.95 (ns)/F = -0.91 (ns)
Pastizal" herbaceous habitat (n = 2)	1.000 +/- 0.500	0.004 +/- 0.004	-
Llanos (n = 3)	0.000	0.000	-
Choco Darien rainforest (n = 1)	-	0.000	-
South East Amazon rainforest (n = 1)	-	0.000	-
Upper Amazon rainforest (n = 22)	0.978 +/- 0.189	0.009 +/- 0.005	D = -0.58 (ns)/F = -3.92 (*p *= 0.05)
Dry tropical forest (n = 4)	1.000 +/- 0.177	0.001 +/- 0.001	D = -0.75 (ns)/F = -2.37 (*p *= 0.01)

*Number refers to Figures 2 & 3

### Demographic history

We explored lowland tapir demographic history across the same three different groupings of samples: (*1*) phylogenetic; (*2*) geographic, and *(3) *ecogeographic. In the phylogenetic grouping, we found significant negative values for Fu's Fs (Fs = -11.26, *p *≤ 0.0001) and Tajima's D (D = -1.55, *p *≤ 0.05) for clade IV, indicating population expansion (Table 3). This was mirrored by the inference by Bayesian skyline plot (BSP) of a two-fold population size increase occurring 15-20 Ky BP in this clade (data not shown). In clade I, only Fu's Fs (Fs = -3.01, *p *≤ 0.05) was significantly negative. In the geographic grouping, we observed significant negative values for Fu's Fs test (Fs = -4.56, *p *= 0.001) and the Tajima's D (D = -1.61, *p *≤ 0.01) only for the south Amazonia group. In the ecoregional grouping, significant negative values were observed with Fu's Fs test only, in the Upper Amazon (Fs = -3.92, *p *= 0.05) and in the southern dry forests (Fs = -2.37, *p *= 0.01).

### Phylogeography

SAMOVA was used to identify maximally differentiated genetic groupings and identify potential phylogeographic breaks. At *K *= 2, the sampling sites from northwest of the continent (Figure 1: sites 2, 4, 6, and 7), were grouped separately from all other sampling sites (*Φ_CT_*= 0.24, *p *≤ 0.0001). At *K *= 3, the groupings were (*i*) western samples: Colombian Amazon (sites 2 and 7), Western Brazilian Amazon (site 3), Ecuadorian Amazon (site 4), Peruvian Amazon (site 6), (*ii*) northern samples: French Guiana (site 1), Venezuelan Llanos (site 8), and western Colombia (site 10); and (*iii*) southern samples: Bolivia (site 9), southern Peru (site 5), Argentina (site 12), and Brazil (site 11) (*Φ_CT_*= 0.29, *p *≤ 0.0001). At *K *= 4, the French Guiana sampling site (site 1) is grouped separately from the two other northern sampling sites (sites 8 and 10; *Φ_CT_*= 0.32, *p *≤ 0.0001). At *K *= 5 to 8, sampling sites from the Upper Amazon region are separated according to river basin separations (Napo, Amazon, Ucayali and Marañón rivers). At *K *> 8, Bolivian (site 9) and Argentinean (site 12) samples are grouped separately.

**Figure 4:**
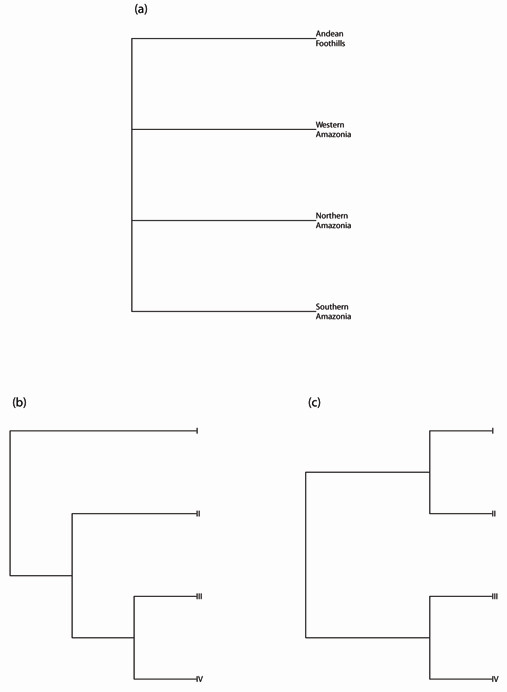
**Phylogeographical hypotheses**: (*i*) the null hypothesis of allopatric divergence within the four main geographic regions (haplotypes grouped by sampling site location irrespective of recovered phylogenetic relationship); and two alternative hypotheses: (*ii*) divergence between clades I and II (as identified from phylogenetic relationships), separating lowland western Amazonia (clade II) from Andean foothills (clade I), followed by divergence of clades III and IV from clade II, leading to the colonization of regions north and south of the Amazon river, respectively; and, (*iii*) split from a lowland western Amazonia ancestral population leading to the colonization of regions north and south of the Amazon river, followed by a later split of lowland western Amazonia and Andean foothills populations.

To test the different proposed biogeographic hypotheses we compared a null model of allopatric divergence among the four geographic areas with colonization models based on different effects of gradient and river-barrier mechanisms of divergence (Figure 4). The reconstructed gene genealogy had an observed Slatkin and Maddison (1989) *s *= 4. For both alternative hypotheses, the null model was rejected at a *p *≤ 0.01 under a wide variety of generation steps and effective population sizes (Table 4).

**Table 4:** Inferred distribution of *s *values [119] calculated under a complex biogeographic hypothesis when compared to a null hypothesis of allopatric differentiation.

Generation*	Effective Population Size (*Ne*)	Inferred *s *(99% CI)
1,250	10,000	19.04 (14 - 23)
1,250	1,000,000	21.116 (15 -25)
375,000	10,000	14.93 (12 - 16)
375,000	1,000,000	17.84 (13 - 21)

^a ^assuming a generation time of 8 years, which gives 10,000 yr BP and 3.1 My BP for 1,250 and 375,000 generations, respectively.

S values are obtained from 1,000 trees simulated under a null model of allopatric divergence (scenario *b *in Figure 4). Similar values were obtained for scenario *c *in Figure 4. Trees were simulated under neutral coalescent with no migration under varying generation times and effective population sizes. Observed *s *= 4.

## Discussion

The role of Quaternary climatic and geological events in promoting divergence and increasing rates of speciation is the subject of intense debate [1-3]. In the Neotropics, the phylogeography and population history of mammals have been investigated in several taxa, including bats [55], carnivores [44,45,56,57], primates [36,37,58], rodents, and marsupials [59-61]. Here, we aimed to contribute to this discussion by exploring the evolutionary history, and population genetic structure and dynamics of the lowland tapir, a habitat generalist and widely dispersed species whose ancestors likely arrived in the continent during the early Quaternary [19]. In doing so, we specifically attempted to estimate time of divergence and identify climatic and geological events that potentially contributed to shaping lowland tapir biogeography, and aimed to provide data relevant for the conservation of this increasingly threatened species.

### Estimating divergence time among populations

On average, mitochondrial DNA mutates at a faster rate than nuclear DNA [62], resulting in it becoming a convenient tool for reconstructing the recent history of populations and species [5]. We applied both the novel approach of Ho and colleagues [63] and the more traditional method based on dating interspecific nodes using fossil data [64] to estimate the mutation rate, and thus the timing of divergence of South American tapir clades. Mutation rate estimates were an order of magnitude higher with the former than with the latter method, and resulted in significantly different mean estimates of *tMRCA*, as seen by the lack of overlap in tree height 95% HPD (0.1 - 0.4 and 0.8 - 4.0 My BP, respectively). Such differences have been reported and discussed elsewhere [65-67] and are generally attributed to differences in intra- and interspecific rates of mutation [64,68]. Consequently, we are not able to *a priori *judge the relative importance of climatic or geological events happening in the late Pleistocene versus early Pleistocene in shaping lowland tapir history.

In order to decide which estimate is more accurate, we would need to test the hypothesis that intra- and interspecific mutation rates have converged [68]. In the case that convergence has occurred, applying fossil data to inform the time of divergence of interspecific tree nodes is expected to result in accurate estimates of the mutation rate. If convergence has not occurred, then fossil calibration is expected to significantly underestimate the mutation rate [63] and the method of Ho and colleagues [63] is then expected to result in more accurate estimates of the mutation rate. Unfortunately, without ancient DNA samples to specifically measure the substitution rate, it is not possible to test the convergence hypothesis. Nevertheless, we believe that other lines of evidence indicate that convergence between intra- and interspecific mutation rates has not occurred. In particular, it has been argued that intra- and interspecific rates of mutation in vertebrates should converge within 2 My of two lineages becoming separated [68], setting a maximum time for convergence to occur. It has also been demonstrated for Adélie penguins, a species with similar generation time to lowland tapir, that convergence of intra- and interspecific rates has not been reached after 44 Ky of two lineages becoming separated [69], thus setting a minimum age of separation before convergence is expected to occur.

An examination of the data available for lowland tapirs contained within the above period highlights an unexplained gap between previously published molecular data and fossil evidences. Previous molecular work estimated that lowland tapirs emerged in the early Pleistocene, soon after their ancestors crossed the Panamana Isthumus [18], which would suggest that convergence between mutation rates has likely occurred. However, an examination of the current fossil record seems inconsistent with this observation. The fossil record includes a number of tapir species described for the period *ca*. 2 My BP throughout South America, particularly in Argentina and Uruguay, but none are classified as *T. terrestris *[15,24]. Instead, the earliest unambiguous fossil described as *T. terrestris *is dated between 80-88 Ky BP [15,24]. This means there is an almost 2 My gap between the earliest described lowland tapir fossil and the time of emergence of lowland tapir previously estimated [18].

The observed incongruence could be either due to too sparsely studied fossil record, although this is not likely due to the wide distribution of tapirs and the extensive paleontological studies [14-17,19,23-29,70], or, to a large previous overestimate of the age of lowland tapir. This later alternative is supported by the similarity of divergence estimates of this study with those of Neotropical carnivores whose ancestors also arrived during the Great American Biotic Interchange [45,57,71]. Thus, it seems more parsimonious to accept that the ancestor to modern lowland tapirs arose around 0.19 and 0.57 My BP (median = 0.35 My BP; 95% HPD 0.19-0.57 My BP), as estimated without calibration of an external node. This would place greater importance on climatic and/or geological events towards the end of the Pleistocene in driving Neotropical diversity than previously proposed [18].

Our conclusions, nonetheless, are based solely on one mitochondrial gene and relatively small sample size, and thus are subjected to large coalescent and sampling variance. Nevertheless, our data suggest novel hypotheses about the history of lowland tapir, and therefore, the potential mechanisms acting to shape Neotropical diversity, which can be further tested using multiple nuclear genes and additional samples.

### Lowland tapir biogeography

Our statistical phylogeography analyses allowed us to reject a null model of allopatric divergence across four major biogeographic regions of the Amazon, as suggested for other taxa [30]. However, it did not distinguish between the two alternative hypotheses of population biogeography (Figure 4). Sampling multiple nuclear genes across additional loci will allow for more accurate estimates of the species' tree and possibly distinguish between the hypotheses. Nevertheless, the rejection of the null hypothesis suggests that lowland tapir history may be complex, and potentially includes separate colonization and population expansion events.

Phylogenetic reconstruction inferred two deeply divergent lowland tapir clades in western Amazonia (clades I and II), with mountain tapirs diverging from one of these lineages. The role of western Amazonia in lowland tapir history has remained a mystery largely because of the paucity of described fossils for the region. A recent report characterized at least three species of tapir occurring in sympatry in the Acre and Rondônia region [15], which led to the hypothesis that the region is a center of origin for tapirs [24]. Our phylogeographic analysis, along with the observation of high genetic diversity in this region (Table 3), supports this scenario, suggesting a similar history as that reported in primates [37,58], carnivores [56], and rodents [72], and adds to reports asserting that western Amazonia has played an important role in divergence and speciation in the Neotropics [32,49,73].

What has led the western Amazon to be a region of high diversity is still unclear, and will likely only be elucidated with additional data on the region's geology. The lack of obvious geographical barriers undermines hypotheses of allopatric divergence to explain the observed structure and diversification in western Amazonia [31]. An alternative hypothesis is that the altitudinal gradient of the Andean foothills along with Pleistocene glacial cycles [51,74] has lead to parapatric divergence among tapir lineages, a model of divergence also proposed for other taxa in western Amazonia and Andean Foothills [e.g., [30]]. At the surface, this hypothesis has support from the adjacent distributions of mountain and lowland tapirs along an elevational gradient from lowland forests to sub-tropical montane habitats [46]. Here, Bayesian analysis indicates little support for lowland and mountain tapir reciprocal monophyly, favoring a paraphyletic relationship. This suggests three scenarios: first, divergence occurred over a relatively short period of time and we are not able to capture the actual relationship with our data [75]; second, mountain tapirs speciated by paraphyly; and third, mountain tapirs are an ecotype of lowland tapir, similar to what is observed in reindeer [76]. In all cases, mountain and lowland tapirs are sister taxa, suggesting a gradient model of divergence.

The results are consistent with phylogeographic patterns reported for other species in the same region [e.g., [71]], thus supporting the scenario that external factors (e.g., climatic or geological) were significant in shaping the history of taxa in this region [77]. However, external mechanisms have been shown to be unnecessary to explain genetic biogeographic structure, as the intrinsic nature of the genealogical process alone can produce significant spatial structure in genetic variation [78,79]. In this regard, future work should concentrate in testing whether independent sections of the genome have similar biogeographic signatures to conclusively rule out stochastic genealogical factors in determining observed structure in genetic diversity [79].

The spatial distribution of the other *cytb *clades (clades III and IV) suggests that the regions north and south of the Amazon River are occupied largely by independent lineages derived from those largely found in western Amazonia. Two main dispersal scenarios can be suggested to account for this pattern. First, assuming that western Amazonia is the origin of dispersal, two separate and independent migration events led to the colonization of the two regions. Alternatively, one lineage colonized one area (e.g., clade III comes out of western Amazonia to colonize north Amazonia), and subsequently originated a new wave of colonization that occupied the second area across the Marajó Archipelago (e.g., clade IV comes out of north Amazonia to colonize south Amazonia, possibly via island hopping). In both cases, and assuming extrinsic factors, a barrier to gene flow is required to avoid admixture and thus dilution of the observed genetic structure, as the geographic boundaries of the lineages' distributions overlap along the Amazon River. Although lowland tapirs are well known swimmers, the river has been reported to be a barrier to jaguars (*Panthera onca*), another similarly large and capable swimmer [45]. Thus, it is plausible that the river is the barrier predicted to account for the current geographic distribution of these two lineages.

Finally, clade IV, which includes all samples from south of the Amazon region (Bolivia and Argentina), a number of haplotypes found in Peru, haplotypes from the Rio Negro (north of the Amazon), and one haplotype from Bolivia that is shared with French Guiana, has an unusually wide geographic distribution when compared to haplotypes of the other three clades. This pattern is generally interpreted as resulting from rapid-range expansion [1], a hypothesis that is supported by the BSP, and Fu's and Tajima's tests. Although the geographic origin of this expansion remains an open question, the expansion is estimated by BSP to have started around the end of the last glacial maximum (LGM), assuming that intra and interspecific rates of mutation have not converged. This would suggest that the LGM significantly reduced lowland tapir population size, similarly to what has been reported for carnivores [71] and ungulates [80]. Alternatively, the expansion also coincides with the extinction of the Neotropical herbivore megafauna, and thus a lessening of interspecific competition may have allowed lowland tapir populations to expand into previously unavailable habitat.

The biogeographical history of the lowland tapir, therefore, is not completely resolved. Future work should explore the potential roles of western Amazonia, the Amazon River, and the LGM in shaping lowland tapir history and biogeography. Although external factors may not have contributed entirely to the observed structure [78,79], comparative phylogeography through the observation of similar patterns across taxa [71,80] provides an indication that more than stochastic coalescent and sampling effects are contributing to the distribution of genetic variation in this species.

### Population diversity, structure and conservation implications

Lowland tapirs are experiencing rapid decline in the Brazilian Atlantic forest and in the Colombian and Venezuelan llanos, and local extinctions have already been reported in Argentina, and southern and eastern Brazil [13]. This decline is attributed to habitat loss and subsistence hunting, and has prompted a "vulnerable" listing for the species in the IUCN Red List [52] and the development of a conservation action plan [81]. However, a key element to any successful action plans is a clear understanding of a species' population history, dynamics, and structure [82-84]. Even though we only examined one mtDNA locus, which can limit our conclusions, our results begin to elucidate such an understanding, providing valuable information for tapir conservation. First, a recent comprehensive status assessment based on habitat sustainability has assigned a low probability of long-term survival for peripheral populations (i.e., Colombian and Venezuelan llanos and southern dry forests groups) [13]. For these same populations, we find lower levels of haplotypic diversity (Table 3) than observed in other areas. This could be due to the reported population decline, but it is also plausible that they have lower genetic diversity because they are at the periphery of the range [85]. In either case, such populations are most likely to harbor unique diversity, as suggested by the SAMOVA results, and thus be important in terms of conserving genetic diversity [79].

Second, we find little support for the use of ecoregions in delimiting areas of lowland tapir conservation priority. South American ecoregions have been defined on the basis of species richness, beta-diversity, and endemism [86]. Tapirs inhabit a wide range of habitats, and thus occur in a number of ecoregions, from savannas and woodlands to lowland and lower montane forests, and as a generalist herbivore they have a broad diet, feeding opportunistically on a wide variety of plants and fruits [87,88]. Studies on jaguars [45], crab-eating foxes [57] and now lowland tapirs indicate that population genetic structure of large neotropical mammals does not necessarily mirror ecoregion boundaries, undermining the utility of these regions to predict Evolutionary Significant Units (ESUs) and Management Units (MUs) [89] for large neotropical vertebrates. Instead, we observed geographic overlap in four phylogenetic clades, precluding delimitation of ESUs; and regions occupied by several independent lineages, making MU status inappropriate. Nevertheless, western Amazonia is an important area for tapir conservation due to its lineage endemism and representatives of other lineages.

## Conclusion

In this study, we derive a number of novel hypotheses to explain lowland tapir biogeography and history. The strengths of these hypotheses include that they complement the fossil record, mirror patterns reported for sympatric taxa, and outline specific alternatives to explain the observed spatial distribution of lineages. In particular, we propose that mountain and lowland tapirs are native to South America, and mountain tapirs may have speciated from the lowland tapir by paraphyly along the steep environmental gradient provided by the Andean foothills in western Amazonia. Differing from previous molecular work but similar to the fossil record, we propose that divergence among lowland tapirs occurred during the late Quaternary, possibly as a consequence of periods of glaciations resulting in significant changes to habitat via cooling, desiccation, or dynamic changes to the river basins [70]. We also propose that independent dispersal events led to the colonization of the regions north and south of the Amazon river and these lineages have remained largely separated due to the barrier that the river represents. Finally, our data suggest that a population expansion occurred after the LGM. However, a more thorough examination based on nuclear genes and additional sampling is required to obtain more accurate estimates of the *tMRCA *and to test if the observed geographic structure is indeed a result of extrinsic factors. More accurate estimates will also put us in a better position to judge if geological and climatic effects have shaped lowland tapir genetic diversity, either in the late Pleistocene or earlier. Finally, similar to other large Neotropical mammals, the lowland tapir exhibits low levels of genetic structuring at the continental scale. From a conservation perspective, our data questions the utility of ecogeographic regions in establishing conservation priorities. Instead, we see western Amazonia and periphery populations emerge as an important harbors of both older and younger lineages, and potentially unique diversity respectively. Further studies across species and habitats at large spatial scales may assist in identifying evolutionary regions across taxa that may be better suited for conservation planning in the Neotropics.

## Methods

### Sampling and DNA sequencing

Genomic DNA was extracted with a standard phenol-chloroform protocol [90] from 12 hair and 33 tissue from animals killed by hunters or captured for ecological studies across the lowland tapirs' distribution including Colombia, Venezuela, Brazil, Ecuador, Peru, French Guiana, Bolivia and Argentina. In addition, we included three hair samples of mountain tapir (*T. pinchaque*) from Colombia (Figure 1, Table 1).

Two overlapping fragments of the target sequence *cytb *were amplified by polymerase chain reaction (PCR) using the primers L7 - CB2 and CB1 - H6. H6 and L7 were designed for Perissodactyls [91], and CB1 (5'-GCCCTTATCCTCTCTAGTTTG-3') and CB2 (5'-AATCGGAGGACAACCAGTTG-3') were designed for this study. Amplification proceeded for 30 cycles of 94°C for 0.5 min, 52°C for 1 min, and 72°C for 1 min. Amplified products were 1,079 bp for L7/CB2 and 313 bp for CB1/H6; both strands were directly sequenced using the PCR primers. Sequence alignments were obtained using MEGA 4.0 [92] and manually checked. Both nucleotide and amino acid sequences were checked for irregularities that could indicate nuclear homologs [93]. All new sequences were deposited in GenBank (accession numbers GQ259910 to GQ259957). We chose *cytb *because data resulting from genetic variability of mitochondrial DNA are commonly used to study species' natural history and dispersal patterns [44,45,57,94,95] and are adequate markers to investigate Evolutionarily Significant Units (ESUs) and Management units (MUs) [96,97]. Because the mitochondrial genome is haploid and maternally inherited, stochastic lineage sorting is expected to progress more rapidly than for nuclear genes. Thus, incomplete sorting is less of a concern for mitochondrial than for nuclear loci, making them ideal for estimating species' trees of closely related taxa [98] and investigating budding speciation [99].

### Phylogenetic analysis and divergence time estimates

Multiple approaches were used to estimate the phylogenetic relationships among the observed haplotypes. First, we estimated the haplotype network using the full set of sequences of *Tapirus terrestris *and *T. pinchaqu*e, using Network 4.5.0. http://www.fluxus-engineering.com and the Median Joining network algorithm [100].

To reconstruct the species gene tree, we used DNASP 5.1 [101] to identify all unique haplotypes in our sample. The gene genealogy was inferred by maximum parsimony and maximum likelihood approaches using the implementations of PAUP [102] and RAxML, respectively, of the CIPRES portal http://www.phylo.org/sub_sections/portal. We also generate a UPGMA tree using $SC:MESQUITE $ESC:[103] based on pairwise distances calculated using an F84 model of nucleotide substitution [104] using haplotypes unique to each sampling site. A strict consensus was used to obtain the final topology across ≤ 100 recovered trees from each method. Finally, we used a Bayesian coalescent approach, implemented in BEAST 1.4.7 [105], using the same parameters outlined below for estimating time of divergence of lowland tapir clades. Estimates of the time to the most recent common ancestors (*tMRCA*) for the different clades were inferred with BEAST 1.4.7 using an approach with calibration based on the age of fossil records [64] and an approach based on independent inferences of Tapiridae *cytb *mutation rates [63] in order to accommodate recently debated issues about molecular dating of relatively recent phylogenetic splits [66-68].

In the first approach, *cytb *sequences from Malay tapir (accession number: AF145734) and several Rhinocerotidae: *Dicerorhinus sumatrensis *(AJ245723), *Diceros bicornis *(X56283), *Ceratotherium simum *(Y07726), *Rhinoceros sondaicus *(AJ245725), *R. unicornis *(X97336) were used as outgroups. Using MRMODELTEST 2.2 [106] we determined that the GTR model of nucleotide substitution with gamma distributed rate variation among sites and nine rate categories (GTR+Γ) was the most suitable nucleotide substitution model for our full sequence set. Instead of using a fixed substitution rate, we imposed monophyly for each species and set strong priors on the nodes corresponding to the most recent common ancestors of Dicerotina and Ceratomorpha. Specifically, we set the prior to a normal distribution with mean (standard deviation) of 17.1 My BP (± 2.5) and 46.7 My BP (± 3.7), respectively. These dates are based on both fossil [107,108] and genetic evidence [91]. The harmonic mean of the model likelihood, f(X/Mi), corresponding to the stationary phase, was compared between molecular clock strategies [109]: relaxed, constant, uncorrelated lognormal, uncorrelated exponential, using the 2LnB10 equation to calculate Bayesian factors (BF) in TRACER 1.4.1 [110]. A BF > 10 provided a strong support for a relaxed molecular clock. Two independent runs (20,000,000 generations with the first 2,000,000 discarded as burn-in and parameter values sampled every 100 generations) with a coalescent prior assuming population exponential growth and a relaxed molecular clock with uncorrelated branch evolutionary rates sampled from an exponential distribution [111] were combined.

In the second approach, we did not use outgroups and external calibration points, instead we set a uniform prior on the substitution rate (0.015 - 0.035 substitutions/site/million years), which encompasses the variation in substitution rate observed for *cytb *in Perissodactyls [54]. Additionally, we set the tree height prior to an exponential distribution with mean of 2.5 and offset of 0.0014, which means tree height can be anything from 14 Ky BP to 9.5 My BP, but puts larger weight on tree heights ≤ 3 My BP. This prior effectively incorporates our uncertainty about the origins of the extant South American tapir species. An offset of 14 Ky BP is the minimum date for which fossils of extant species have been unambiguously identified [15] and 9.5 My BP is the age of the first *Tapirus *fossil in North America [8], however previous estimates suggest that lowland and mountain tapir diverged soon after entering South America in the late Pliocene [18]. As described above, we determined that the HYK+I model was the most suitable nucleotide substitution model for our 48 sequences; and using BF, we determined that the sequences were behaving in accordance with a strict molecular clock rather than a relaxed clock, and that a constant population size prior had a better fit than an exponential growth prior. For each model we sampled the posterior distribution 40,000,000 times, logging every 1,000 steps for a total of 40,000 data points, the first 10,000 data points were discarded as burn-in.

In all cases, model results were visually inspected using TRACER V 1.4.1 to ensure proper mixing of the MCMC and that all parameters had ESS values above 1,000. The final tree for each approach, including divergence estimates and their 95% highest posterior densities (HPD), were computed in TREEANNOTATOR 1.4.5. Posterior probability values were used to assess the degree of support of each node on the tree, and we reported the maximum sum of clade credibility tree [111].

### Population genetic diversity and structure

Population structure was investigated according to three hypothesized scenarios: (*i*) populations were defined according to strongly supported clades identified in the gene tree reconstruction; (*ii*) populations were defined according to four wide geographic regions, that are partially suggested by phylogenetic clades: Andean foothills, western Amazonia, north Amazonia, and south Amazonia including Bolivia and Argentina (Table 1); and (*iii*) populations were defined according to ecogeographic units (Table 1, [13]), which combine ecoregions [86] and regional habitats used for identification of priority areas for biodiversity conservation [53].

For each of these scenarios, DNASP 5.1 [101] and ARLEQUIN 3.1 [112] were used to examine nucleotide site polymorphism, haplotypic diversity (*H_d_*), and nucleotide diversity (*π*) of each group of samples. Genetic variation was partitioned and fixation indices were estimated using an analysis of molecular variance (AMOVA), as implemented in ARLEQUIN. Pairwise *Φ_CT_*significance tests were also calculated using ARLEQUIN.

### Demographic history

Two approaches were used to examine the historical patterns of population growth in the lowland tapir. Under the assumption of neutrality, deviations in Tajima's D [113] and Fu's Fs [114] were used to test for a recent population expansion or bottleneck [115]. Second, Bayesian skyline plots (BSP) [116] were used to visually inspect changes in *N_e_*over time. BSPs were constructed in BEAST 1.4.7 [105] using the same priors as described above plus eight fixed stepwise changes in *N_e_*. We sampled the posterior distribution with an MCMC of 80,000,000 generations, logged every 1,000 steps for a total of 80,000 data points, the first 20,000 were discarded as burn-in. The scaled effective population size was converted to the effective population size, *N_e_*, assuming a generation time of 10 years [81] and the mutation rate calculated in BEAST (section above).

### Phylogeography

A spatial analysis of molecular variance, as implemented in the program SAMOVA 1.0 [117], was used to define partitions of sampling sites that are maximally differentiated from each other without any *a priori *assumption about population structure. The method is based on a simulated annealing procedure that maximizes the proportion of genetic variance that can be explained by differences between groups of populations, assessed with the among group genetic variation (*Φ_CT_*) coefficient as estimated by AMOVA [118]. Analyses were based on 100 simulated annealing steps with number of groups (*K*) increasing from 1 to 12, allowing us to identify the clustering of samples that yielded the largest, and most significant, *Φ_CT_*for a given *K*.

To further examine the biogeographic history and the mechanisms involved in lowland tapir divergence, we used MESQUITE 2.72 [103] to calculate Slatkin and Maddison's *s *[119] in the recovered gene tree and two hypothetical scenarios, which are suggested by the haplotype network and the observed gene genealogy (Figure 4): (*i*) a split between Andean foothills and lowland western Amazonia (gradient hypothesis), followed by an eastward and southern expansion event split into two fronts by the Amazon River (river hypothesis); (*ii*) an eastward expansion event split into two fronts by the Amazon River (river barrier), and split between Andean foothills and western Amazon plain (gradient). Both scenarios are associated with a pattern of isolation-by-distance, but differ in the relative timing of the events. To test the significance of the observed *s *for the observed gene genealogy under each of these hypotheses, we generated null distributions for the test statistic by simulating 1,000 gene trees under a neutral coalescent process without migration under a null hypothesis of allopatric fragmentation and calculated *s *for each gene tree under each of our alternative population tree hypotheses [120]. To reject the null hypothesis of allopatric fragmentation, in which samples were grouped according to the geographic scenario described above, we should observe a significantly smaller *s *between the recovered gene tree and our hypotheses than between gene trees simulated under an allopatric fragmentation and our hypotheses. We chose the allopatric fragmentation scenario because it is regarded as the principal mode of diversification in vertebrates [49], and allows us to specifically test for a refugia model of diversification, as these grouping reflect putative Amazonia refugia [31]. Finally, as the coalescent process is dependent on the effective population size and number of generations [121], we tested 10,000 years and 3,100,000 years to *tMRCA*, and *N_e_*of 10,000 and 1,000,000, for a total of four scenarios. Ten thousand years and 3,100,000 years are tested because they refer to the end of the last glacial maximum and to the emergence of the Panama Isthmus, respectively. The effective population sizes were chosen because they reflect our uncertainty about tapir census population sizes [13], and how they might translate into effective population sizes. In addition, these values encompass the N_e _estimate based on BSP [116] by our inference of the mutation rate obtained from BEAST across all four clades.

## Authors' contributions

BdT collected samples, carried out the molecular work, analysed the data, and drafted the original manuscript. AGS, MRG and AL collected samples, extensively contributed to data analysis, and helped to draft the manuscript. AT, OR, MA, VQ, CPM, MT and CP contributed to sample collection, generated DNA sequences in their respective laboratories, and contributed to the interpretation of the data. All authors read and approved the final manuscript.
